# Multiplex-Polymerase Chain Reaction for Detecting Microdeletions in The Azoospermia Factor Region of Y Chromosome in Iranian Couples with Non-Obstructive Infertility and Recurrent Pregnancy Loss

**DOI:** 10.22074/ijfs.2018.5162

**Published:** 2017-10-14

**Authors:** Afsaneh Mojtabanezhad Shariatpanahi, Hassan Ahmadnia, Adam Torkamanzehi, Mahnaz Mansouri Torshizi, Mohammad Amin Kerachian

**Affiliations:** 1 Department of Biology, University of Sistan and Baluchestan, Zahedan, Iran; 2 Medical Genetics Research Center, Mashhad University of Medical Sciences, Mashhad, Iran; 3 Department of Urology, Ghaem Hospital, Mashhad University of Medical Sciences, Mashhad, Iran; 4 Novin Infertility Center, Mashhad, Iran; 5 Department of Medical Genetics, Faculty of Medicine, Mashhad University of Medical Sciences, Mashhad, Iran

**Keywords:** Infertility, Multiplex Polymerase Chain Reaction, Y Chromosome

## Abstract

**Background:**

Approximately 15% of couples are infertile with the male factor explaining approximately 50% of the cases. One of the main genetic factors playing a role in male infertility is Y chromosomal microdeletions within the proximal long arm of the Y chromosome (Yq11), named the azoospermia factor *(AZF)* region. Recent studies have shown there is a potential connection between deletions of the *AZF* region and recurrent pregnancy loss (RPL). The aim of this study is to examine this association by characterizing AZF microdeletions in two infertile groups: in men with non-obstructive infertility and in men with wives displaying RPL.

**Materials and Methods:**

In this is a case-control study, genomic DNA was extracted from 80 male samples including 40 non-obstructive infertile men, 20 males from couples with RPL and 20 fertile males as controls. Multiplex polymerase chain reaction was used to amplify 19 sequence tagged sites (STS) to detect AZF microdeletions. Differences between the case and control groups were evaluated by two-tailed unpaired t test. P<0.05 were considered statistically significant.

**Results:**

Only one subject was detected to have Y chromosome microdeletions in *SY254, SY157* and *SY255* among the 40 men with non-obstructive infertility. No microdeletion was detected in the males with wives displaying RPL and in 20 control males. Y chromosome microdeletion was neither significantly associated with non-obstructive infertility (P=0.48) nor with recurrent pregnancy loss.

**Conclusion:**

Performing Testing for Y chromosome microdeletions in men with non-obstructive infertility and couples with RPL remains inconclusive in this study.

## Introduction

Y chromosome is the shortest chromosome in the human genome. It has the least number of genes among all human chromosomes (1). The human Y chromosome is necessary for human sex determination, and male germ cell development and maintenance (2). Of the 60 Mb length of the Y chromosome, 3 Mb belongs to pseudoautosomal regions (PAR1 and PAR2 on the Yp and Yq respectively) and 57 Mb to a nonrecombining region (NRY). The NRY region can be classified to heterochromatic and euchromatic regions. The euchromatin contains all of the known genes in the Y chromosome. The euchromatic regions on the Y are about 23 Mb consisting of 8 Mb on the short arm and 14.5 Mb on the long arm (1, 3). Genes located on the euchromatic region of the proximal long arm of the Y chromosome (Yq11), named *azoospermia factor (AZF) *region, plays an essential role in spermatogenesis (4, 5). Recent studies have suggested that the AZF region cause male infertility and also recurrent pregnancy loss when it is disrupted (6, 7).

### Y Chromosome and male infertility

Approximately 15 percent of couples are infertile with the male factor being responsible for approximately 50% of the cases. It is defined as a multifactorial syndrome encompassing a wide variety of disorders (8). In about 50-60% of male infertility cases, the etiology can be identified, however, when the cause is unknown, it is referred to as idiopathic infertility (9). A significant proportion of idiopathic male infertility is associated with azoosperima or severe oligozoospermia, which may be due to genetic alterations. Nevertheless, the underlying etiology is still poorly understood (10).

Recent studies have shown that both genetic and environmental factors are involved in the reduction of reproductivity in males. The main genetic factors in male infertility are Y chromosomal microdeletions within the Yq11 region and somatic chromosomal abnormalities. After Klinefelter syndrome, Y chromosomal microdeletion is the most frequent cause of male infertility (11) and the second most frequent genetic cause of spermatogenic failure (12). The microdeletions in the *AZF* region occur in infertile men (13). Studies have shown that the *AZF* region is deleted in about 13% of men with non-obstructive azoospermia and in 7 to 10% of men with oligozoospermia (14). Testicular tissue sections of azoospermic men with these Yq11 aberrations showed intense spermatogenesis disruption. This suggested that there is an essential function of *AZF* for differentiation and proliferation of human male germ cells (15). The *AZF* region consists of four sub-regions, namely *AZFa, AZFb, AZFc* and *AZFd* (14, 16, 17). Each of these regions are associated with a particular testicular histology, and a number of candidate genes have been found within these regions (13). Deletions in the *AZF* region occur as six classical types of Yq deletions consist of *AZFa, AZFb, AZFc, AZFbc, AZFabc* and partial *AZFc* (3) as described in Table 1.

**Table 1:** Genotype-phenotype correlation of AZF regions (3, 18)


Deletion	Deletions are known to correspond to:

*AZFa* deletion	Complete *AZFa* deletions: severe testicular phenotype, SCOS and spermatogenic arrest
	Partial AZFa deletions: extremely rare
AZFb deletion	Complete *AZFb* deletions: spermatogenic arrest
	Partial AZFb deletions: variable phenotypes from hypospermatogenesis to SCOS extremely rare
*AZFc* deletion	Complete *AZFc* deletions: variable phenotype which may range from mild oligospermia to azoospermia and SCOS
Partial *AZFc* deletion	Variable phenotypes from hypospermatogenesis to the SCOS
*AZFbc* deletion	SCOS/spermatogenic arrest
*AZFabc* deletion	SCOS


*AZF*; Azoospermia factor and SCOS; Sertoli cell-only syndrome.

### Y Chromosome and recurrent pregnancy loss

Recurrent pregnancy loss (RPL), recurrent miscarriage or habitual abortion is the occurrence of three or more consecutive pregnancies that terminate through miscarriage before fetus viability (for instance, 24 weeks of gestation). About 1% of couples trying to have children are affected by recurrent miscarriage (19). RPL is a multifactorial condition with several etiologic factors including genetic abnormalities of the parents, endocrinologic, anatomic, hematologic and immunologic abnormalities along with nutritional, infectious and environmental factors (20, 21). The most commonly accepted etiology of RPL is maternal, however, most cases are classified as idiopathic, with no identifiable cause in either partner (20, 22). The repetitive pregnancy loss in some couples plus the high percentage of idiopathic RPL indicate that the underlying causes of RPL needs to be investigated (23). Mutations including small deletions, duplications and substitutions cannot be detected by cytogenetic analysis. These genetic abnormalities may thus account for a large number of miscarriages with unknown causes (24).

New evidence indicates that male factors may play a major role in RPL (25). Sperm integrity is required for fertilization, sperm-egg interactions and early embryonic development. Sperm quality affects the ability of the embryo to reach the blastocyst phase and develop into implantation. Paternally expressed genes control the proliferation and invasiveness of trophoblast cells, and also placental proliferation (7). The cause of pregnancy loss in approximately 50% of women with RPL remains unexplained despite many investigations (26). Recent studies have shown there is a potential connection between deletions of the *AZF* region and RPL (7, 26, 27). In a study by Dewan et al. (7), analysis of male partners in couples with RPL showed 82% of the men had at least one *AZF* microdeletion. Studying Y microdeletion is thus crucial in understanding and predicting the outcome of future pregnancies, and making informed decisions regarding treatment such as assisted reproductive technology (ART). Therefore, the aim of this study was to detect Y chromosomal microdeletions in men with non-obstructive infertility and in men having spouses with RPL by using a multiplex PCR design.

### Materials and Methods

This was a case-control study. It consisted of three groups. The first group comprised 40 infertile men (azoospermic and severe oligozoospermic) aged 20-53 years old, who were referred to the Ghaem General Hospital and Novin Infertility Clinic in Mashhad, Iran, between September 2012 and September 2013. All patients in this group had primary infertility with normal karyotype and absence of obstructive azoospermia. The second group consisted of 20 men aged 17-42 years from couples with history of three or more consecutive idiopathic miscarriages, all of whom were referred from the High Education Center of Jahad Daneshgahi, Mashhad, Iran, from 2011 to 2013. In this group all men and their spouses had a normal karyotype. Other causes of pregnancy loss including infectious disease, and psychological, uterine anatomic and endocrine disorders along with immunologic and haemostatic changes were excluded. A group of 20 healthy men aged 25-42 years from couples with at least one live birth and no history of miscarriage was considered as the control group (third group).

### Statistical analysis

Differences in microdeletion frequency were examined by two-tailed unpaired t test. A P<0.05 was considered statistically significant. Demographic data of the patient and control groups were also analyzed. All the above were computed using the SPSS-V11 software. Our study was approved by the Ethics Committee of Mashhad University of Medical Sciences. An informed consent was obtained from each individual participating in this study.

### Y microdeletion multiplex polymerase chain reaction detection assay

Genomic DNA was extracted from 3 ml of peripheral blood lymphocyte samples using a standard salting-out method. Isolated DNA was stored at -20°C. Following DNA extraction, *AZF* microdeletions were screened by multiplex polymerase chain reaction (PCR). Nineteen sequence- tagged sites (STSs) within the long arm of the Y chromosome were selected to cover *AZFa, b, c* and proximal *AZFc (AZFd)* regions. For each participant, *18 STS in AZFa (sY81, sY86, sY182), AZFb (sY121, sY124, sY127, sY128, sY130, sY133, sY134, SYPR3), AZFc (sY157, sY208, sY242, sY254, sY255) and AZFd (sY145, sY152)* sub-region were typed. The primers were combined into five sets for multiplex PCR for the purpose of determining the presence of all 19 sequence-tagged sites by performing five parallel PCR amplifications from multiplex A to E.

Multiplex reaction A amplified *SY81, SY130, SY157, SY182, SY254, B amplified SYPR3, SY127, SY208, SY242, C amplified SY121, SY128, SY145, SY255, D amplified SY124, SY133, SY152, SMCX* and E amplified *SY14(SRY) SY86, SY134 ZFX/Y*. Multiplex D contained a control primer pair amplifying a fragment of the X-linked SMCX locus and multiplex E contained a control primer pair amplifying a unique region present on both the Y and X chromosomes (*Zinc Finger Protein of Y and X chromosomes ZFX/ZFY*). These control primer pairs were used as internal controls to check amplification of DNA and also the integrity of the genomic DNA sample used. Finally, the multiplex E reaction included a primer pair amplifying a region of SRY (*sex-determining region of the Y*). The presence of the short arm of the Y chromosome (Yp) was tested with *STS SY14*, located within *SRY*. The *SRY* was examined to confirm the sex of the sample donor.

PCR was carried out in a total volume of 15 μl containing 150 ng of genomic DNA, 1X PCR buffer, 2 mM of MgCl2, 1 unit of Taq DNA polymerase (Genet Bio, South Korea), 0.2 mM of dNTP mix and 4 pmol of each primer. The cycling conditions were an initial denaturation at 95°C for 5 minutes, followed by 35 cycles of denaturation at 95°C for 30 seconds, annealing for 30 seconds at 59°C in multiplex A, at 57°C in multiplex B and at 56°C in multiplex reactions C, D, E, and extension at 72°C for 35 seconds, followed by a last extension at 72°C for 5 minutes and a cooling step at 4°C. The PCR products were separated on a 3.5% agarose gel using 1X TAE. PCR bands were visualized using DNA Green Viewer and under ultraviolet light.

## Results

**Fig.1:**
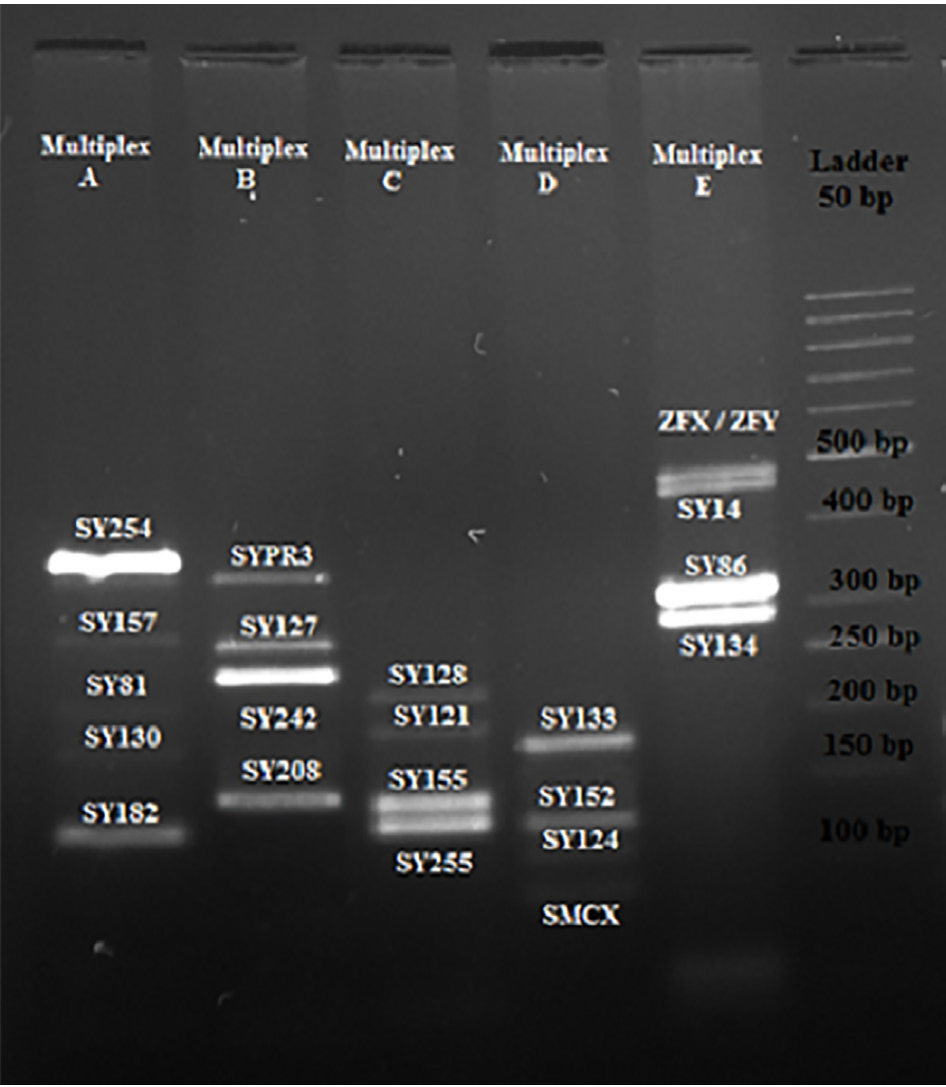
Results of the multiplex reactions A, B, C, D and E in male partners of women with recurrent pregnancy loss (RPL). Polymerase chain reaction (PCR) fragments were separated on a 3.5% agarose gel. Lane 1; Multiplex A, Lane 2; Multiplex B, Lane 3; Multiplex C, Lane 4; Multiplex D, and Lane 5; Multiplex E. Molecular weight marker (50 bp ladder).

**Fig.2:**
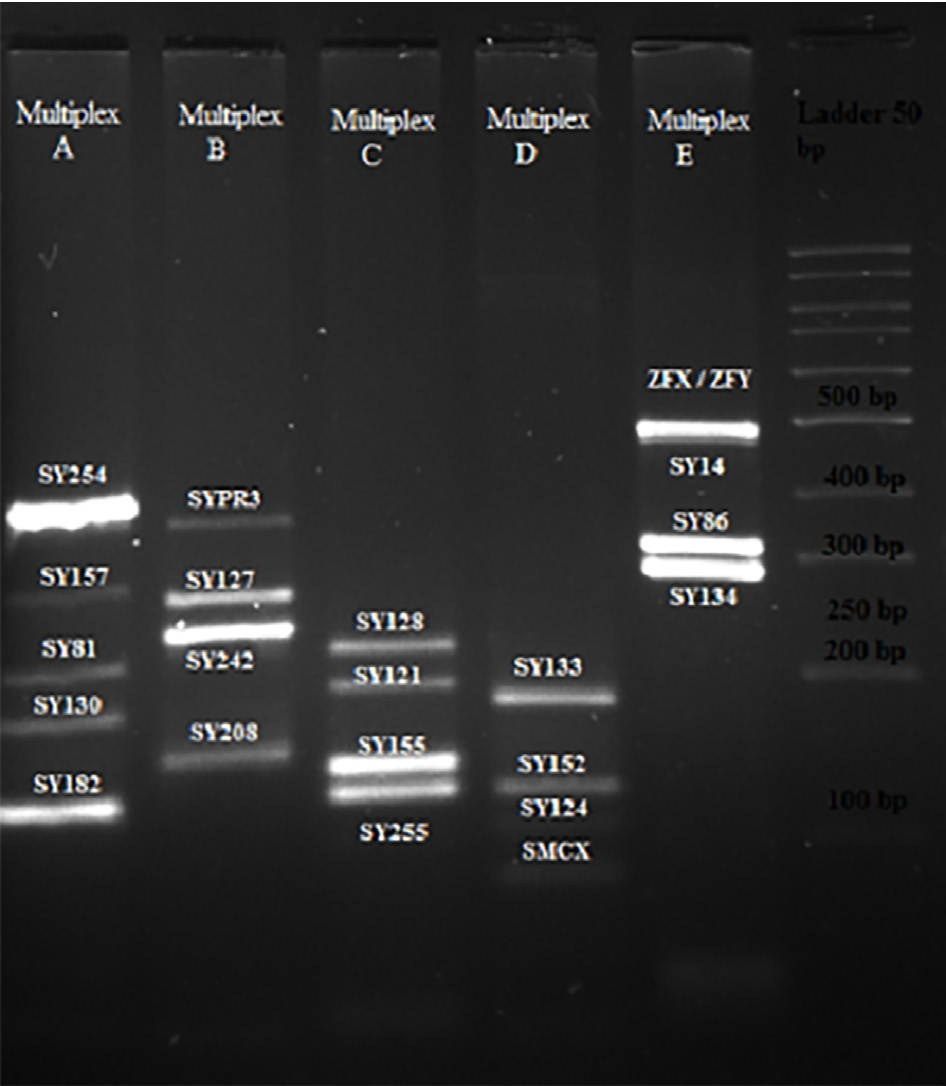
Results of the multiplex A, B, C, D and E in control group. Polymerase chain reaction (PCR) fragments were separated on 3.5% agarose gel. Lane 1; Multiplex A, Lane 2; Multiplex B, Lane 3; Multiplex C, Lane 4; Multiplex D, and Lane 5; Multiplex E. Molecular weight marker (50 bp ladder).

**Fig.3:**
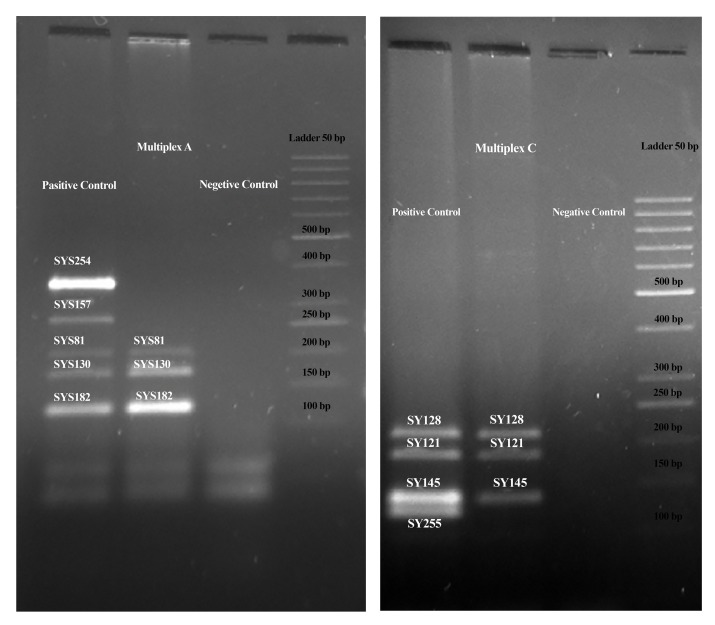
Detection of partial *AZFc* deletion in an infertile male patient. A. The microdeletions observed were in Multiplex A (*SY254* and *SY157*) and B. In Multiplex C (*SY255*). Polymerase chain reaction (PCR) fragments were separated on a 3.5% agarose gel. Lane 1; Fertile control, Lane 2; Infertile man, and Lane 3; Negative control. Multiplex A reaction contained SY81 (209 bp), SY130 (173 bp), SY157 (290 bp), *SY182* (125 bp) and *SY254* (380 bp), and multiplex C reaction contained *SY121* (190 bp), *SY128* (228 bp), *SY145* (143 bp) and *SY255* (124 bp). Molecular weight marker (50 bp ladder).

No microdeletion in the *AZFa, AZFb, AZFc* and *AZFd* sub-regions was observed in male partners of women with RPL (Fig .1) and men in the control group (Fig .2). Among the 40 infertile men, only one subject (2.5%) had microdeletions in multiplex reactions A and C, indicative of a microdeletion in the *AZFc* region (Fig .3A, B). *AZF* microdeletion was neither significantly associated with nonobstructive infertility (P=0.48) nor with RPL.

## Discussion

The AZF region was originally identified by Tiepolo and Zuffardi (28). These microdeletions are thought to be pathogenetically involved in some cases of male infertility who have azoospermia or severe oligozoospermia (4). Although chromosomal abnormalities in sperms of infertile men may lead to RPL (29), microdeletions in the AZFc region of the Y chromosome may have an important function in embryo “competency” or in maintaining gestation. This has led to Y-chromosome AZFc microdeletion testing in RPL cases when no other explanation for RPL is known (7).

The Y chromosome is extremely rich in repetitive sequences, organized in amplicons forming eight palindromes. Most of the genes deleted in infertile men are located in the palindromic regions of the Yq and are exclusively expressed in the testes (3, 13). Since AZF microdeletions usually include more than one gene, the role of a single AZF gene cannot be specified and thus unclear. Gene-specific deletions removing a single gene has been only reported in the AZFa region (30). In our study, a single infertile man (2.5%) had microdeletion in the AZFc region (partial AZFc deletions), which displays a lower frequency of AZF microdeletions than other reports in Iran (5, 31-33).

Y chromosome microdeletions were neither found in the male partners of women experiencing RPL nor in the control group. Although this finding is in agreement with the results obtained by Ghorbian et al. (24), it does not support the results of Soleimanian et al. (27) who detected Y chromosome microdeletions in male partners of women with RPL. This discrepancy could be explained by the small sample size, which is a limitation of the current study. In addition, differences in genetic background of the population studied here and the typing of different sets of STS used in different studies may explain the differences in the frequency of AZF microdeletions. Adjusted sample size and use of identical sets of STS could lessen the variation in results.

## Conclusion

We showed Y chromosome microdeletions were not associated with non-obstructive infertility and recurrent pregnancy loss in our population study. Thus, this study is not supporting to test for AZF microdeletions in these two groups.
